# The uptake of influenza vaccination for the 2013/2014 season in Germany

**DOI:** 10.17886/RKI-GBE-2017-124

**Published:** 2017-12-13

**Authors:** Christina Poethko-Müller, Birte Bödeker

**Affiliations:** Robert Koch Institute, Department of Epidemiology and Health Monitoring, Berlin

**Keywords:** SEASONAL INFLUENZA, VACCINATION, THE WINTER SEASON OF 2013/2014, HEALTH MONITORING, GERMANY

## Abstract

Seasonal influenza (flu) is an acute viral disease that occurs every winter in Germany and is referred to as the ‘flu wave’. The Standing Committee on Vaccination (STIKO) at the Robert Koch Institute recommends annual vaccination for people who are at greater risk of disease-related complications, this includes men and women aged 60 or above. In this target group, 48.1% of women and 48.7% of men who participated in the GEDA 2014/2015-EHIS study reported that they had been vaccinated against influenza during the 2013/2014 winter season. However, there were marked differences according to region. Vaccination rates decreased over time. Moreover, although the European Commission has been calling for a vaccination rate of at least 75% among the elderly from the 2014/2015 influenza season onwards, it is unclear to what extent this rate can actually be achieved. However, rates can be improved by doctors providing advice and recommending vaccination to their patients.

## Introduction

Seasonal influenza (flu) is an acute viral disease that affects the population of the northern hemisphere almost every winter. It occurs as a cluster that lasts for a number of weeks and is referred to as a ‘flu wave’. Influenza is typically characterised by the sudden onset of illness with a high fever, dry cough and pain in the muscles, neck and/or limbs [1]. Depending on the particular virus strain and a person’s level of immunity, a large proportion of people with an infection may only suffer mild symptoms and in many cases the disease may even occur without any recognisable symptoms [2]. On average, an influenza infection lasts between five and seven days, but it can persist for longer, especially when combined with certain risk factors. Severe cases frequently result in lung complications such as pulmonary infections [1, 3]. It is important to differentiate between colds, which are often referred to as ‘influenza-like illnesses’, and seasonal influenza. Colds result in much milder symptoms than influenza and are caused by different pathogens. Whereas influenza is triggered by influenza viruses, colds are caused by more than 30 different pathogens, including RSV, rhinovirus and coronaviruses. In contrast, seasonal influenza results from viruses such as Influenza A(H3N2), Influenza A(H1N1)pdm09 and Influenza B. Importantly, different virus strains are predominant in different years and the strength of each flu wave can vary considerably. According to calculations by the Robert Koch Institute’s Influenza Working Group (AGI), between one and five million influenza-related visits are made to doctors annually; in years with a severe flu wave this figure can be significantly higher (see also the focus article on respiratory diseases in issue 3/2017 of the Journal of Health Monitoring). When severe flu waves do occur, as was the case with the 2012/2013 winter season, approximately 30,000 influenza-related hospital admissions and 20,000 deaths can be linked to influenza. In contrast, the flu wave that occurred during the 2013/2014 winter season was considered comparatively mild: it led to just 3,000 extra hospital admissions and no increase in mortality [4, 5].

**GEDA 2014/2015-EHIS**

**Data holder:** Robert Koch Institute

**Aims:** To provide reliable information about the population’s health status, health-related behaviour and health care in Germany, with the possibility of a European comparison

**Method:** Questionnaires completed on paper or online

**Population:** People aged 18 years and above with permanent residency in Germany

**Sampling:** Registry office sample; randomly selected individuals from 301 communities in Germany were invited to participate

**Participants:** 24,016 people (13,144 women; 10,872 men)

**Response rate:** 26.9%

**Study period:** November 2014 - July 2015

**Data protection:** This study was undertaken in strict accordance with the data protection regulations set out in the German Federal Data Protection Act and was approved by the German Federal Commissioner for Data Protection and Freedom of Information. Participation in the study was voluntary. The participants were fully informed about the study’s aims and content, and about data protection. All participants provided written informed consent.

More information in German is available at www.geda-studie.de

Since influenza viruses are constantly changing, a single vaccination is not enough to prevent the disease. Influenza vaccination is aimed at preventing influenza infections, influenza-related complications and possible deaths. The Standing Committee on Vaccination (STIKO) at the Robert Koch Institute (RKI), therefore, recommends that people with a higher risk of disease-related complications are vaccinated annually against the influenza viruses in current circulation. Vaccination is particularly important for people aged 60 or above, for people with chronic diseases, women in the second or third trimester of pregnancy or when an underlying disease that has been present since the beginning of pregnancy causes increased health risks, and for people who are at a higher risk of disease due to occupational exposure, such as medical staff [6]. In this latter case, vaccination not only protects medical professionals, it also protects their patients. The considerable disease burden associated with influenza has led the European Commission to strive for an influenza vaccination rate of at least 75% among the elderly from 2014/2015 onwards [7].

## Indicator

In the GEDA 2014/2015-EHIS study, data on the uptake of influenza vaccination during the 2013/2014 winter season was gathered using a self-reporting questionnaire that the respondents completed on paper or online. The respondents were asked ‘Were you vaccinated against flu in the run-up to the 2013/2014 winter season?’ The recall period ranged from 8 to 23 months depending on when the questionnaire was completed.

The analyses undertaken for the GEDA study are based on information gathered from 23,339 participants aged 18 or over (12,815 women; 10,524 men) with valid information on the uptake of flu vaccination for the 2013/2014 winter season. The calculations were carried out with a weighting factor that corrects the sample for deviations from the population structure in Germany (as of 31 December 2014) with regard to gender, age, type of municipality and education. ‘Municipality type’ refers to the degree of urbanisation in a particular area and corresponds to the regional distribution in Germany. The International Standard Classification of Education (ISCED) was used to classify the participants’ educational and occupational qualifications [8]. A statistically significant difference between groups is assumed to have been demonstrated when 95% confidence intervals do not overlap.

A detailed description of the methodology used for GEDA 2014/2015-EHIS can be found in Lange et al. 2017 [9] as well as in the article German Health Update: New data for Germany and Europe in issue 1/2017 of the Journal of Health Monitoring.

## Results and discussion

25% of participants aged 18 or above reported that they been vaccinated against flu for the 2013/2014 winter season; the rates for women (25.6%) and men (24.5%) were very similar. Despite the fact that around 75% of people aged 60 or above need to be vaccinated annually, 48.1% of women and 48.7% of men in this age group reported that they had been vaccinated against flu in the 2013/2014 winter season. No association between vaccination and educational status were identified (Table 1).

The influenza vaccination rate among people aged 60 or above (the target group) has decreased in recent years. Compared with the results of the GEDA studies conducted in 2009, 2010 and 2012, vaccination rates among people aged 60 or above declined from 57% in 2008/2009 to 48% in the winter season of 2013/2014 [10]. This reduction is also very clear from accounting data held by the Associations of Statutory Health Insurance Physicians (KV) [11]. Moreover, the annual vaccination rate among people aged 60 or over (of under 50%) identified in these studies remains significantly lower than the rate recommended by the European Commission (75%) [7], without any indication of increased influenza vaccination uptake [10, 11].

**Figure 1:**
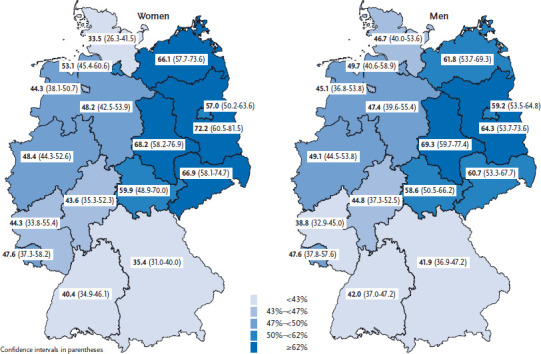
The rate of vaccination against seasonal influenza (flu) in the 2013/2014 winter season in women and men aged 60 or above, by federal state (n=1,706 women; n=1,743 men) Source: GEDA 2014/2015-EHIS

There are clear regional differences in the uptake of influenza vaccination. Among people aged 60 or above the highest rates are found in the new German federal states (as was the case in previous years). This further demonstrates the greater acceptance of vaccination in the new federal states [13]. Among the target group aged 60 or above, the highest rates of vaccination during the 2013/2014 influenza season were achieved in Brandenburg, Saxony-Anhalt, Saxony and Mecklenburg-Vorpommern. In contrast, the federal states with the lowest vaccination rates are located in the extreme south (Bavaria) and the extreme north (Schleswig-Holstein), with rates of 38.4% and 39.8% respectively (Figure 1). The accounting data held by the KV show that regional differences in influenza vaccination rates are not only limited to the federal state level, but even occur at the district level. In fact, these data demonstrate extreme differences at the district level with vaccination rates in 2013/2014 ranging from between 13.5% and 65% [12].

The GEDA and KV data show broadly consistent results with regard to changes in vaccination rates over time as well as in terms of the differences between federal states. However, the estimated 48% vaccination rate identified among the elderly in the 2013/2014 influenza season by the GEDA study is significantly higher than the rate calculated from the accounting data (38%) [11, 12]. A telephone-based study identified a rate of 49.4% (95% confidence interval 44.6-54.2) for the 2013/2014 influenza season, which is similar to the result from GEDA 2014/2015-EHIS [14]. Two factors may have contributed to the higher rates identified by these studies: people who are less health conscious may also be less willing to participate in health studies, and respondents may tend to provide socially desirable answers to studies based on questionnaires and interviews.

There are important reasons why people do not ensure that they are vaccinated against influenza. First, flu is not always viewed as a serious illness that poses a danger to health; at the same time, people are sometimes doubtful about the safety and efficacy of vaccination. However, people do not always reject vaccination outright; rather, they sometimes forget to arrange an appointment for vaccination or do not do so for other reasons [14-17]. The GEDA 2014/2015-EHIS study demonstrates that only about a quarter of people aged 60 or over have never been vaccinated against influenza; as such, a large proportion of the people who were not vaccinated against flu during the 2013/2014 season may not be opposed to influenza vaccination in principle. In Germany as well as in other countries, doctors play an important role in the decisions leading to vaccination [14, 18, 19]. However, a 2009 survey of doctors’ practices demonstrates that doctors do not necessary encourage all particularly vulnerable groups to ensure that they are vaccinated against influenza [20]. At the same time, Influenza vaccination rates among medical staff are also still low in Germany and vary between 26% and 73% [20, 21] depending on the survey and the influenza season in question. The results presented here demonstrate the great opportunity that exists to increase the annual influenza vaccination rate among the elderly. Target group-specific measures aimed at medical professionals who are responsible for vaccination could be a useful means of doing so. This would involve raising awareness about this issue, ensuring that it forms part of (further) training, increasing payments for vaccination and related consultations, and making billing processes more simple. Finally, more effort should be made to stress the importance of influenza vaccination among people aged 60 or above and to increase vaccination rates. This could include introducing a statutory responsibility to recall patients for vaccination as part of active patient care, bonuses, and providing information about the benefits of vaccination to individuals and society as a whole, and about the serious complications that can be caused by vaccine-preventable diseases.

## Key statements

- Less than 50% of people aged 60 or over are vaccinated against influenza.
- The vaccination rate of 75% among the elderly, which the European Commission has been striving for since the 2014/2015 influenza season, has yet to be achieved.
- Flu vaccination coverage is very different depending on the region in question.
- Medical advice, including recommendations by doctors to vaccinate, is an important way of increasing vaccination rates.

## Figures and Tables

**Table 1:** The rate of vaccination against seasonal influenza (flu) in the 2013/2014 winter season by gender, age and educational status (n=12,815 women; n=10,524 men) Source: GEDA 2014/2015-EHIS

Women	%	(95% CI)	Men	%	(95% CI)
**Women (total)**	**25.6**	**(24.3-26.9)**	**Men (total)**	**24.5**	**(23.3-25.7)**
**18-29 Years**	9.5	(8.0-11.3)	**18-29 Years**	9.9	(8.2-12.0)
Low education	10.7	(7.2-15.6)	Low education	16.4	(11.9-22.1)
Medium education	9.4	(7.7-11.5)	Medium education	7.7	(6.0-9.9)
High education	7.0	(4.8-10.2)	High education	7.9	(5.2-11.8)
**30-44 Years**	11.6	(10.2-13.3)	**30-44 Years**	11.7	(10.3-13.4)
Low education	7.3	(4.5-11.7)	Low education	9.9	(6.1-15.8)
Medium education	12.1	(10.2-14.4)	Medium education	12.1	(10.0-14.5)
High education	13.0	(10.7-15.8)	High education	11.5	(9.4-14.0)
**45-59 Years**	19.4	(17.8-21.2)	**45-59 Years**	19.7	(17.9-21.7)
Low education	20.6	(16.5-25.4)	Low education	19.9	(15.2-25.5)
Medium education	18.8	(16.9-20.9)	Medium education	20.2	(17.8-22.9)
High education	20.4	(17.8-23.3)	High education	18.7	(16.2-21.4)
**≥60 Years**	48.1	(45.8-50.4)	**≥60 Years**	48.7	(46.6-50.8)
Low education	51.1	(47.5-54.6)	Low education	46.5	(41.5-51.7)
Medium education	46.4	(43.4-49.5)	Medium education	50.5	(47.4-53.7)
High education	46.0	(41.8-50.3)	High education	46.4	(43.4-49.4)
**Total (women and men)**	**25.0**	**(24.0-26.1)**	**Total (women and men)**	**25.0**	**(24.0-26.1)**

CI=confidence interval
